# Prevalence of Avoidant/Restrictive Food Intake Disorder (ARFID) in children with and without food allergy

**DOI:** 10.1111/pai.70393

**Published:** 2026-07-07

**Authors:** Daniela Ciciulla, Jennifer J. Koplin, Michele Yeo, Mimi L. K. Tang, Kirsten P. Perrett, Rushani Wijesuriya, Vicki McWilliam, Rachel L. Peters

**Affiliations:** ^1^ Department of Pediatrics University of Melbourne Melbourne Victoria Australia; ^2^ Population Allergy Murdoch Children’s Research Institute Parkville Victoria Australia; ^3^ Centre for Food and Allergy Research (CFAR) Murdoch Children’s Research Institute Parkville Victoria Australia; ^4^ Child Health Research Centre University of Queensland South Brisbane Queensland Australia; ^5^ Department of Adolescent Medicine Royal Children’s Hospital Parkville Victoria Australia; ^6^ Brain and Mind Research, Murdoch Children’s Research Institute Parkville Victoria Australia; ^7^ Department of Allergy and Immunology Royal Children’s Hospital Parkville Victoria Australia; ^8^ Allergy Immunology Murdoch Children’s Research Institute Parkville Victoria Australia; ^9^ Clinical Epidemiology and Biostatics Unit Murdoch Children’s Research Institute Parkville Victoria Australia

**Keywords:** ARFID, eating disorders, feeding disorders, food allergy, food allergy anxiety, IgE‐mediated, prevalence

## Abstract

**Introduction:**

Emerging research suggests those with food allergy may have a higher risk of eating and feeding disorders; however, data on the prevalence of Avoidant/Restrictive Food Intake Disorder (ARFID) among individuals with food allergy is limited. We examined whether possible ARFID in 10‐year‐old children was more common in children with or without food allergy.

**Method:**

The HealthNuts study recruited 5276 one‐year‐olds across Melbourne, Australia who were followed‐up at ages 4, 6, and 10 years. Food allergy was assessed at each age with skin prick tests, clinical history, and/or oral food challenges. The Food Allergy Quality of Life Parent Form (FAQLQ‐PF) capturing food allergy anxiety was completed for those with suspected food allergy. The ARFID assessment tool ‘Eating Disorders in Youth Questionnaire (EDY‐Q)’ was completed by children participating in their age 10 assessment after 10 April 2019.

**Results:**

A total of 951 children completed the EDY‐Q, of whom 102 had a current food allergy. The prevalence of possible ARFID was similar among children with current food allergy, 23% (95% CI 15–32) compared to without, 21% (95% CI 18–24). When considering food allergy anxiety (reported on FAQLQ‐PF) as a symptom of possible ARFID (a variant not captured by EDY‐Q), an additional 10 children (of 102) with food allergy may meet the criteria for possible ARFID but were missed by the EDY‐Q.

**Conclusion:**

A similar proportion of children with and without food allergy were identified by the EDY‐Q of being at risk of ARFID. The EDY‐Q may lack sensitivity to detect ARFID in individuals with food allergy as it does not capture fear of allergic reactions. Newer ARFID tools incorporating fear of allergic reactions have since become available. Future research using these tools may provide more precise prevalence estimates among individuals with food allergy.

**Abbreviations**
AAI: Adrenaline Autoinjector
ARFID: Avoidant/Restrictive Food Intake Disorder
ARFID‐FAA: Avoidant/Restrictive Food Intake Disorder inclusive of Food Allergy Anxiety
BPFAS: Behavioral Pediatric Feeding Assessment Scale
DSM: Diagnostic and Statistical Manual of Mental Disorders
EDY‐Q: Eating Disorders in Youth Questionnaire
FAA: Food Allergy Anxiety
FAQLQ‐PF: Food Allergy Quality of Life Questionnaire Parent Form
GOS: Great Ormond Street
HREC: Human Research Ethics Committee
ISSAC: International Study of Asthma and Allergies in Children
OFC: Oral Food Challenge
PARDI‐AR‐Q: Pica, ARFID, and Rumination Disorder Interview ARFID Questionnaire
SES: Socioeconomic Status
SPT: Skin Prick Test

**Key message**

Emerging evidence suggests eating and feeding disorders are more common in individuals with food allergy; however, inconsistent nomenclature makes it difficult to define. This study found that the prevalence of symptoms of ARFID was similar in children with and without food allergy. However, some ARFID screening tools may under‐detect symptoms of ARFID in individuals with food allergy because they do not capture fear of aversive consequences specific to food allergy such as food allergy anxiety. Clinicians should be aware that symptoms of ARFID may present in children with food allergy and consider referring children exhibiting symptoms of eating and feeding difficulties for additional assessment and management.

## 1 INTRODUCTION

Food allergy is a growing public health concern. In Australia, approximately 6% of 10‐year‐old children have IgE‐mediated food allergy and 40% have at least one allergic condition (food allergy, hay fever, asthma, eczema), which often co‐occur.^1^ Food allergy management requires exclusion diets and if not supported adequately, these can contribute to nutritional deficiencies, reduced growth, poor quality of life and parental anxiety.^2^, ^3^, ^4^, ^5^, ^6^ In addition, dietary and psychosocial restriction beyond what is necessary to maintain allergy safety can manifest, further exacerbating the inherent nutritional risk and psychosocial burdens that are present with food allergy.^4^, ^7^, ^8^ These patterns could have previously been labeled as a specific phobia or anxiety disorders, however, they are also consistent with symptoms of Avoidant/Restrictive Food Intake Disorder (ARFID).^9^ ARFID was first defined in 2013, in the 5th edition of the Diagnostic and Statistical Manual of Mental Disorders (DSM5) and revised in 2018.^10^, ^11^ Currently, ARFID is defined as avoidance or restriction of food intake without body image or weight distortion but rather due to [1] lack of interest in eating/food, [2] sensory aversion or [3] fear of aversive consequences of eating (e.g., choking, vomiting, gastrointestinal discomfort and allergic reactions). Diagnosis requires that the restriction results in at least one of the following manifestations: [1] growth/weight impact, [2] significant nutritional deficiency, [3] dependence on enteral feeding or supplementation or [4] marked interference with psychosocial functioning. As noted in the DSM5, ARFID can co‐occur with medical conditions, including food allergy, if the eating disturbance exceeds that routinely associated with the condition or persists following resolution of the medical condition.^10^, ^12^

If not detected early or untreated, ARFID can lead to health complications as a consequence of a severely impacted diet.^9^, ^13^, ^14^, ^15^, ^16^, ^17^, ^18^ A meta‐analysis on global ARFID prevalence in children and adults found a pooled prevalence of 4.5% (95% CI 0.1–10.68), noting significant heterogeneity (*I* ^2^ = 99.6%), high publication bias and low‐moderate quality of evidence.^19^ Our recent systematic review reported that the prevalence of ARFID among children and adults with food allergy (IgE‐mediated and eosinophilic oesophagitis) ranged from 5% to 63% again noting high heterogeneity, risk of bias and limited studies (*n* = 4).^20^ The only study on individuals with IgE‐mediated food allergy reported that ARFID occurred in 63% (*n* = 54); however, this is likely an overestimate as some of the sample was also recruited from a feeding clinic.^21^ Identifying individuals at risk of ARFID and establishing accurate prevalence estimates are essential for informing service demand, advocating for resources, and informing research priorities. We aimed to determine the prevalence of possible ARFID in 10‐year‐old children with and without food allergy, in Melbourne, Australia. Additionally, among children with food allergy, we describe the demographic and clinical characteristics of those with possible ARFID.

## 2 METHODS

### 2.1 Study design

**FIGURE 1:**
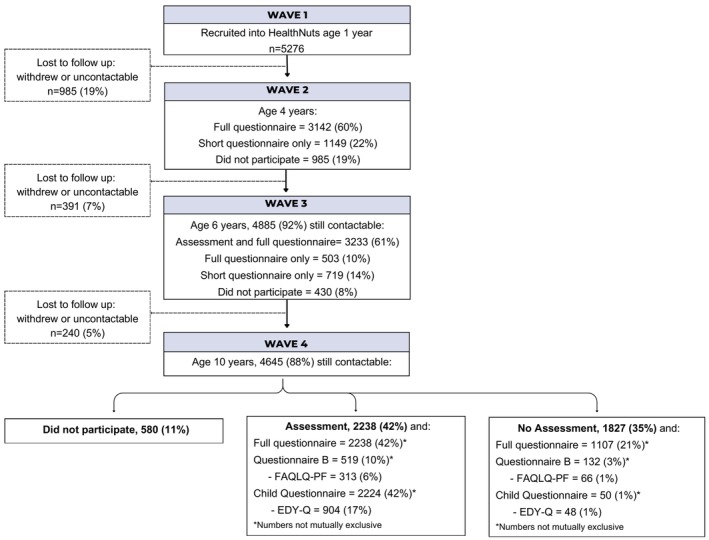
Participation in the longitudinal HealthNuts study of allergic diseases. Figure adapted from Peters et al.^1^ Participants who declined to participate at one age and had not withdrawn were contacted to participate at later waves. Percentages in parentheses refer to the proportion of participants in the original cohort who participated. If parents declined to complete the full assessment or questionnaire, a short telephone questionnaire was completed to capture key details on allergy history.

HealthNuts is a prospective longitudinal study of allergic disease; the target population was 1 year. old infants residing in Melbourne, Australia, recruited irrespective of allergy risk. Methods have been described in detail previously (Figure 1).^1^, ^22^, ^23^, ^24^

Briefly, at *Wave 1 (age 1 year)* 5276 infants aged 11–15 months were recruited from council‐run immunization sessions in Melbourne between 2007 and 2011. Infants underwent a skin prick test (SPT) to peanut, egg, sesame, and either cow's milk or shellfish. Those with a SPT wheal size ≥1 mm to peanut, sesame, or egg were offered an oral food challenge (OFC). At *Wave 2 (age 4 years)*, parents/caregivers completed questionnaires and those with new reported food reactions indicative of IgE‐mediated allergy or prior OFCs at wave 1, were invited for a clinic assessment including SPT and OFC to eight common foods (peanut, sesame, egg, milk, wheat, almond, cashew, hazelnut) and any additional foods to which they reported reactions to.^24^ Methods for *Wave 3 (age 6 years)* and *Wave 4 (age 10 years)* were the same. All children were invited to complete an allergy assessment which included SPT to 10 core foods (peanut, sesame, egg, milk, wheat, almond, cashew, hazelnut, soy, and brown shrimp) and anthropometric measurements. Participants were offered OFC to a core food if they had a confirmed food allergy at a previous wave and were still avoiding the food, reported new food reactions indicative of IgE‐mediated food allergy, or were newly SPT positive and were not consuming or had a food reaction. SPT and possible OFC were performed to non‐core foods if a parent reported a reaction to the food consistent with IgE‐mediated food allergy. Food allergy definitions for each age are provided in Table 1.

**TABLE 1:** Definitions used to classify food allergy outcomes in the HealthNuts study. Table adapted from Parker et al.^25^

Timepoint	No food allergy	Definite food allergy	Probable food allergy
Wave 1 Age 1 year	Negative OFC result; or SPT wheal 0 mm.	Positive OFC result and food sensitised; or Food sensitised and a reaction consistent with OFC stopping criteria within the last 1‐2 months.	N/A
Wave 2 Age 4 years	Negative OFC result; or Negative SPT result; or Food sensitised and ingestion history indicative of food tolerance: (i) eaten the index food > 3 times, no recent reactions, and not avoiding the index food; or (ii) negative OFC result at the last wave to the index food.		SPT wheal ≥ 8 mm and no OFC result; or SPT wheal 2 mm and positive OFC result; or Definite food allergy at the last wave and parent‐report of allergist confirmed persistent food allergy; or Parent‐reported food avoidance due to allergy and a reaction consistent with an IgE‐mediated food allergy within the last 12 months.
Wave 3 Age 6 years		Positive OFC result and food sensitised; or Food sensitised, no OFC result and a reaction consistent with an IgE‐mediated food allergy within the last 12 months; or SPT wheal ≥ 8 mm and positive OFC result at the last wave to the index food.	SPT wheal ≥ 8 mm, no OFC result at the current and last wave, no known food tolerance; or Food sensitised, no OFC result and positive OFC result at the last wave; or Food sensitised and a reaction consistent with an IgE mediated food allergy since the last wave.
Wave 4 Age 10 years			

*Note*: Food sensitization age 1 years: SPT wheal ≥ 2 mm and/or sIgE ≥ 0.35 kU_A_/L. Food sensitization age 4 to 10 years: SPT wheal ≥ 3 mm and/or sIgE ≥ 0.35 kU_A_/L. Current food allergy: Definite or probable food allergy at age 10 years. Absence of current allergy was defined as not having allergy to all of the core foods (i.e. if absence of food allergy was missing for one or more of the core foods, or non‐core food allergy outcomes were inconclusive they were excluded from analysis). Ever food allergy: Definite or probable food allergy at age 1, 4, 6 or 10 years. Absence of food allergy ever was defined as not having allergy to any of the tested food at any follow‐up. Missing data was treated as no allergy.

Abbreviations: N/A, not applicable; OFC, oral food challenge; SPT, skin prick test sIgE, specific IgE.

Parents/caregivers completed questionnaires at all follow‐ups. Questionnaires included the “*Full questionnaire*” capturing information on the child's allergy history including validated surveys from the ISAAC study.^26^, ^27^, ^28^ At the 10 year follow‐ups, if parents indicated that their child had adverse food reactions, they also completed “Questionnaire B”. Within Questionnaire B, if parents indicated their child had a current food allergy, the Food Allergy Quality of Life Parent Form (FAQLQ‐PF) was completed.^29^ Children completed a “*Child questionnaire*” at 10 years (Figure 1). The Child questionnaire was provided during assessments except at the time of the Victorian COVID‐19 lockdowns where assessments were paused and children were sent the child questionnaire online. The Eating Disorders in Youth Questionnaire (EDY‐Q), which was added to the Child questionnaire on 10th April 2019, is a screening tool with psychometric validation in 8–13 year olds to detect early onset restrictive eating disturbances characteristic of ARFID.^30^, ^31^ As the EDY‐Q is a screening rather than a diagnostic tool, and conducting diagnostic clinical interviews was not feasible within the scope of this study, definitive ARFID diagnoses could not be established; therefore, this study is limited to reporting on possible ARFID outcomes.

### 2.2 Food allergy definition

*Current food allergy at 10 years*: was defined as having an allergy to any of the core or non‐core foods tested; absence of allergy was defined as not having allergy to all of the core foods (i.e. if absence of food allergy was missing for one or more of the core foods, or non‐core food allergy outcomes were inconclusive they were excluded from analysis).

*Food allergy ever*: was defined as having an allergy to at least one of the foods tested across any of the follow‐ups; absence of food allergy ever was defined as not having an allergy to any of the tested food at any follow‐up. Missing data was treated as no allergy.

*Persistent allergy*: was defined as allergy present at any of the previous follow‐ups that persisted at that age 10 follow‐up.

*Resolved allergy*: was defined as allergy present at any previous follow‐up which resolved by the age 10 follow‐up.

### 2.3 Possible ARFID definitions

The EDY‐Q is a 12‐item screening tool based on DSM5 ARFID diagnostic criteria and the Great Ormond Street (GOS) criteria. Responses are reported on a 7‐point Likert scale (never = 0; always = 6).^31^ The 12 items include eight on selective eating, emotional food avoidance, and functional dysphagia to cover the central variants of ARFID, two items on underweight problems, indicating the failure to meet adequate energy needs, and two items on weight and shape concern as exclusion criteria of ARFID.

The original EDY‐Q scoring criteria for possible ARFID, proposed when the tool was first developed^30^, ^31^ require that at least one of the items on variants of ARFID to be reported at least often (≥4): (variants: disinterest in food OR sensory food avoidance OR fear of choking/vomiting) AND the item on underweight problems reported as ≥4, while weight and shape concern need to be reported less than sometimes (<3) (Table 2). If items on weight and/or shape concern were missing and the questionnaire was otherwise sufficiently completed, the data were treated as if they were ≥3 on these items. As items on weight and shape concern may be sensitive questions particularly when weight or shape cognitions are present, missing responses were conservatively treated as indicative of endorsement of these concerns to minimize the risk of misclassifying individuals as possible ARFID.

**TABLE 2:** Definitions of possible ARFID.

	Definition
Primary definition: Possible ARFID; across the weight spectrum	1. EDY‐Q item on disinterest in food (Q2) **OR** sensory food avoidance (Q12) **OR** fear of choking/vomiting (Q10) reported at least often (≥4), **while** 2. Weight and shape concern (Q6 & Q7) reported less than sometimes (<3).
Alternative definition: Possible ARFID with subjective underweight—Original definition described by Kurz et al.^31^	Based on original scoring of the EDY‐Q proposed by (Kurz et al.^31^); 1. EDY‐Q item on disinterest in food (Q2) **OR** sensory food avoidance (Q12) **OR** fear of choking/vomiting (Q10) reported at least often (≥4) **AND** 2. Underweight problems (Q4) reported ≥4, **while** 3. Weight and shape concern (Q6 & Q7) reported less than sometimes (<3).
New definition derived for this study: Possible ARFID inclusive of food allergy anxiety (ARFID‐FAA)	1. EDY‐Q item on disinterest in food (Q2) **OR** sensory food avoidance (Q12) **OR** fear of choking/vomiting (Q10) reported at least often (≥4) **OR** FAQLQ‐PF item on food allergy anxiety reported at least quite a bit ≥4, **while** 2. Weight and shape concern (Q6 & Q7) reported less than sometimes (<3).

Since the development of the EDY‐Q, the DSM5 diagnostic criteria have been updated to acknowledge that ARFID can occur across the weight spectrum.^11^ Therefore, the primary definition of possible ARFID used in this study removes the requirement for underweight, while the original scoring criteria is used as an alternative definition (see Table 2).

The EDY‐Q does not capture fear of allergic reactions which is a subtype of fear of aversive consequences. To address this, we derived a new definition of possible ARFID that incorporates food allergy related anxiety. Fear of allergic reaction was measured by the FAQLQ‐PF (on a 7‐point Likert scale (not at all = 0; extremely = 6)) through one item: “Because of food allergy my child feels anxious about food”. We applied cut‐off of ≥4 to indicate food allergy anxiety (FAA). This cut‐off was selected pragmatically for consistency with the EDY‐Q scoring approach and was further informed by the World Allergy Organization consensus on Definition of Food Allergy Severity.^32^ To identify children with fear of aversive consequences related to food allergy that EDY‐Q may miss, we defined possible ARFID inclusive of food allergy anxiety (ARFID‐FAA) using our primary definition of possible ARFID, with FAA allowed to count as one of the ARFID variants (Table 2).

### 2.4 Ethics

Consent to participate in the HealthNuts study was provided at each wave by the child's parents or caregivers. Approval to conduct the HealthNuts study was obtained from the Victorian State Government Office for Children (reference no. CDF/07/492), the Victorian State Government Department of Human Services (reference no. 10/07), and the Royal Children's Hospital Human Research Ethics Committee (HREC number 27047 and number 32294).

### 2.5 Statistical analysis

The prevalence of possible ARFID in the age 10 follow up is reported as the observed proportion with 95% confidence interval. The relative difference in odds of having possible ARFID between those with and without food allergy (current or ever) was estimated by logistic regression. Analyses were performed using Stata 18 (StataCorp, College Station, TX).

## 3 RESULTS

### 3.1 Participation

Of 5276 children enrolled in HealthNuts at age 1, 88% (*n* = 4645) were still contactable at age 10 years (Figure 1). The Child Questionnaire was completed by 2274 children, of which 952 recruited after 10th April 2019 completed the EDY‐Q. One participant was excluded due to incomplete EDY‐Q data. Of the 951 participants, 1% (11/951) had missing data from either or both of the EDY‐Q items assessing weight/shape cognitions (ARFID exclusion criteria). The FAQLQ‐PF was completed by 379 participants, and of those, 145 also completed the EDY‐Q. Overall, 2238 had an assessment which included allergy testing. Comparison of demographics from participants and non‐participants at age 10 years has been described previously, with participants more likely to have personal or family history of allergy, an older mother, and higher socioeconomic status (SES).^1^, ^22^ Further, children who completed the EDY‐Q were similar to children who completed the Child Questionnaire prior to EDY‐Q's inclusion, with the exception that a history of food allergy was more common in the EDY‐Q participants (Table S1).

### 3.2 Prevalence of possible ARFID

Using the primary definition, the prevalence of possible ARFID across the weight spectrum was 21% (95% CI 19%–24%; *n* = 204/951). Children with possible ARFID had lower BMI z‐scores than those without (−0.1 and 0.2, respectively); however, there was little difference in deceleration in BMI z scores from age 6 to 10 years. between groups. Possible ARFID was more common in children with allergic comorbidities compared to those without (25% vs. 18%). The difference was largest in hay fever (26% vs. 18%) (Table 3). Other characteristics were similar between children with and without possible ARFID. The prevalence of possible ARFID with subjective underweight was 5% (95 CI 3–6; Table S2).

**TABLE 3:** Distribution of possible ARFID by demographic and clinical characteristics among 10‐year old children.

	No ARFID *n* = 747^a^	Possible ARFID *n* = 204	*p*‐value
Age in years (mean (SD))	10.3 (0.3)	10.30 (0.3)	0.88
Sex			0.94
Male	382 (79%)	104 (21%)	
Female	363 (78%)	100 (22%)	
SES score^b^			0.48
1 (most disadvantaged)	147 (76%)	46 (24%)	
2	151 (81%)	35 (19%)	
3	167 (81%)	40 (19%)	
4	150 (79%)	39 (21%)	
5 (least disadvantaged)	331 (75%)	44 (25%)	
Ethnicity			0.55
Both parents Caucasian	569 (79%)	150 (21%)	
One/both parents aboriginal or Torres Strait Islander	3 (75%)	1 (25%)	
One/both parents Asian	139 (79%)	38 (21%)	
Other	36 (71%)	15 (29%)	
Parental country of birth			0.42
Both born Australia	467 (78%)	131 (22%)	
One/both born south/east Asia	121 (81%)	28 (19%)	
One/both born United Kingdom Europe	89 (82%)	20 (18%)	
Other	69 (73%)	25 (27%)	
Family history of allergy^c^			0.83
No	207 (79%)	55 (21%)	
Yes	540 (78%)	149 (22%)	
Height *z* scores (mean [SD])	0.4 (1.0)	0.3 (1.0)	0.13
Missing	37	12	
BMI *z* scores (mean [SD])	0.2 (1.0)	−0.1 (1.0)	0.0001
Missing	41	12	
BMI percentile			0.001
<5th Percentile	15 (60%)	10 (40%)	
5–85th Percentile	533 (77%)	160 (23%)	
85–95th Percentile	90 (92%)	8 (8%)	
>95th Percentile	68 (83%)	14 (17%)	
Deceleration in BMI *z* score from age 6 to 10 years			0.65
No deceleration	600 (79%)	161 (21%)	
−1 *z* score	40 (73%)	15 (27%)	
−2 *z* scores	2 (67%)	1 (33%)	
−3 *z* scores	1 (100%)	0	
Subjective underweight			<0.001
No	662 (81%)	159 (19%)	
Yes	81 (65%)	43 (35%)	
Current allergic comorbidities^d^			0.014
No	385 (82%)	86 (18%)	
Yes	331 (75%)	110 (25%)	
Current eczema			0.96
No	595 (78%)	167 (22%)	
Yes	101 (78%)	28 (22%)	
Current asthma			0.43
No	642 (79%)	170 (21%)	
Yes	105 (76%)	33 (24%)	
Current Hay fever			0.004
No	486 (82%)	109 (18%)	
Yes	249 (74%)	89 (26%)	

^a^ Of the 951 participants *n* = 11 had missing data from either or both of the EDY‐Q items assessing weight/shape cognitions (ARFID exclusion criteria). This data was treated as if it was a score of ≥3 on these items, therefore meeting ARFID exclusion criteria.

^b^ Socioeconomic status defined using Socioeconomic Index for Areas (Quintiles, 1 = most disadvantages and 5 = least disadvantaged) at age 1 year.

^c^ Includes history of food allergy, asthma, eczema, and hay fever in parents and siblings collected at wave 1 (12 months old).

^d^ Any current eczema, asthma or hay fever: The definitions of current eczema, asthma and hay fever have been previously described by Peters et al.^1^

The prevalence of possible ARFID among 10‐year‐old children with current food allergy was 23% (95% CI 15–32) compared to 21% (95% CI 18–24) in children without food allergy (OR 1.09 (0.67–1.80)). Findings were similar when compared between those with and without food allergy ever, persistent and resolved food allergy, and anaphylaxis ever (Table 4).

**TABLE 4:** Association between food allergy and possible ARFID.

	Possible ARFID %, (95% CI)	Unadjusted Odds ratio	
	*n* = 951	OR (95% CI)	*p*‐value
Food allergy ever			
No (*n* = 767)	21% (18–24)	Ref	
Yes (*n* = 184)	23% (17–29)	1.10 (0.75–1.62)	0.61
Current food allergy			
No (*n* = 771)	21% (18–24)	Ref	
Yes (*n* = 102)	23% (15–32)	1.09 (0.67–1.80)	0.72
Change in egg and peanut allergy over time			
Never egg allergic (*n* = 632)	22% (18–25)	Ref	
Resolved egg allergy (*n* = 109)	23% (16–32)	1.09 (0.67–1.76)	0.74
Persistent egg allergy (*n* = 12)	25% (7–59)	1.22 (0.33–4.55)	0.77
Never peanut allergic (*n* = 693)	22% (19–25)	Ref	
Resolved peanut allergy (*n* = 12)	17% (4–52)	0.73 (0.16–3.37)	0.69
Persistent peanut allergy (*n* = 55)	20% (11–33)	0.91 (0.46–1.81)	0.79
Anaphylaxis ever			
Never food allergy (*n* = 767)	21% (18–24)	Ref	
Food allergy ever without anaphylaxis (*n* = 83)	22% (14–32)	1.03 (0.60–1.80)	0.91
Food allergy ever with anaphylaxis (*n* = 69)	23% (15–34)	1.13 (0.63–2.02)	0.69
Adrenaline autoinjector prescription ever			
Never food allergy (*n* = 767)	21% (18–24)	Ref	
Food allergy ever without prescription (*n* = 94)	24% (17–34)	1.21 (0.73–2.00)	0.46
Food allergy ever with prescription (*n* = 90)	21% (14–31)	1.00 (0.62–1.70)	0.99

*Note*: Ref indicates the reference category for the odds ratios.

### 3.3 Food allergy anxiety and possible ARFID

Incorporating FAA as ARFID variant (ARFID‐FAA) identified an additional 10 (10%) children as having possible ARFID‐FAA, bringing the prevalence of possible ARFID‐FAA among children with food allergy to 32%, 95% CI 24%–42%.

### 3.4 Is ARFID or ARFID‐FAA more common in sub‐groups of children with current food allergy?

Table 5 presents the distribution of possible ARFID and ARFID‐FAA by demographic and clinical characteristics among children with current food allergy. ARFID and ARFID‐FAA were more common in children who reported subjective underweight. Possible ARFID and ARFID‐FAA were more common in children with food allergy who had other allergic conditions, and this was largely driven by hay fever. Possible ARFID‐FAA was higher among children with a history of anaphylaxis (38% vs. 26%) or adrenaline autoinjector (AAI) prescription (36% vs. 20%), compared to those without. Overall, among children with current food allergy, 21/102 reported FAA, with most (76%) classified as possible ARFID‐FAA. The distribution of possible ARFID by demographic and clinical characteristics among children with food allergy ever is provided in Table S3; findings are largely similar; however, possible ARFID prevalence did not differ across anaphylaxis and AAI subgroups.

**TABLE 5:** Distribution of possible ARFID and ARFID‐FAA by demographic and clinical characteristics among children with current food allergy.

	No ARFID *N* = 79	Possible ARFID *N* = 23	*p* value	No ARFID‐FAA N = 69	Possible ARFID‐FAA *N* = 33	*p* value
Sex			0.78			0.88
Male	54 (78%)	15 (22%)		47 (68%)	22 (32%)	
Female	25 (76%)	8 (24%)		22 (67%)	11 (33%)	
Socioeconomic status^a^			0.64			0.61
1 (most disadvantaged)	8 (67%)	4 (33%)		8 (67%)	4 (33%)	
2	15 (83%)	3 (17%)		15 (83%)	3 (17%)	
3	20 (71%)	8 (29%)		17 (61%)	11 (39%)	
4	14 (78%)	4 (22%)		12 (67%)	6 (33%)	
5 (least disadvantaged)	22 (82%)	4 (15%)		17 (65%)	9 (35%)	
Ethnicity			0.30			0.34
Both parents Caucasian	43 (78%)	12 (22%)		35 (64%)	20 (36%)	
One or both parents aboriginal or Torres Strait Islander	0	1 (100%)		0	1 (100%)	
One or both parents Asian	31 (79%)	8 (21%)		29 (74%)	10 (26%)	
Other	5 (71%)	2 (29%)		5 (71%)	2 (29%)	
Parental country of birth			0.60			0.10
Both born Australia	36 (73%)	13 (27%)		28 (57%)	21 (43%)	
One or both born south/east Asia	24 (83%)	5 (17%)		23 (79%)	6 (21%)	
One or both born United Kingdom Europe	8 (89%)	1 (11%)		8 (89%)	1 (11%)	
Other	10 (71%)	4 (29%)		9 (64%)	5 (36%)	
Family history of allergy^b^			0.31			0.14
No	18 (86%)	3 (14%)		17 (81%)	4 (19%)	
Yes	61 (75%)	20 (25%)		52 (64%)	29 (36%)	
Height *z* scores (mean [SD])	0.3 (0.8)	0.6 (0.9)	0.07	0.3 (0.9)	0.4 (0.8)	0.52
BMI *z* scores (mean [SD])	0.1 (1.1)	−0.2 (1.2)	0.21	0.2 (1.1)	−0.2 (1.0)	0.11
Missing	2	‐		2	‐	
BMI percentile			0.13			0.037
< 5th Percentile	2 (50%)	2 (50%)		2 (50%)	2 (50%)	
5–85th Percentile	55 (74%)	19 (26%)		45 (61%)	29 (39%)	
85–95th Percentile	12 (100%)	0		12 (100%)	0	
>95th Percentile	8 (80%)	2 (20%)		8 (80%)	2 (20%)	
Deceleration in BMI *z* score from age 6 to 10 years			0.85			0.73
No deceleration	66 (79%)	18 (21%)		57 (68%)	27 (32%)	
−1 *z* score	6 (75%)	2 (25%)		6 (75%)	2 (25%)	
−2 *z* scores	0	0		0	0	
−3 scores	1 (100%)	0		1 (100%)	0	
Subjective underweight			0.044			0.16
No	69 (81%)	16 (18%)		60 (71%)	25 (29%)	
Yes	10 (59%)	7 (41%)		9 (53%)	8 (47%)	
Current allergic comorbidities^c^			0.082			0.012
No	15 (94%)	1 (6%)		15 (94%)	1 (6%)	
Yes	59 (74%)	21 (26%)		49 (61%)	31 (39%)	
Current Eczema			0.79			0.38
No	46 (77%)	14 (23%)		37 (62%)	23 (38%)	
Yes	23 (74%)	8 (26%)		22 (71%)	9 (29%)	
Current Asthma			0.75			0.65
No	51 (78%)	14 (22%)		45 (69%)	20 (31%)	
Yes	28 (76%)	9 (24%)		24 (65%)	13 (35%)	
Current Hay fever			0.10			0.026
No	33 (87%)	5 (13%)		31 (82%)	7 (18%)	
Yes	46 (73%)	17 (27%)		38 (60%)	25 (40%)	
Anaphylaxis ever			0.51			0.22
No	37 (80%)	9 (20%)		34 (74%)	12 (26%)	
Yes	42 (75%)	14 (25%)		35 (63%)	21 (38%)	
Adrenaline autoinjector prescription ever			0.73			0.13
No	20 (80%)	5 (20%)		20 (80%)	5 (20%)	
Yes	59 (77%)	18 (23%)		49 (64%)	28 (36%)	
Because of food allergy, anxious about food			0.37			0.000
No	62 (81%)	15 (19%)		62 (81%)	15 (19%)	
Yes	15 (71%)	6 (29%)		5 (24%)	16 (76%)	

^a^ Socioeconomic status defined using the Socioeconomic Index for Areas (Quintiles, 1 = most disadvantaged and 5 = least disadvantaged) at age 1 year.

^b^ Includes history of food allergy, asthma, eczema, and hay fever in parents and siblings collected at wave 1 (12 months old).

^c^ Any current eczema, asthma or hay fever: The definitions of current eczema, asthma and hay fever have been previously described by Peters et al.^1^

### 3.5 ARFID‐FAA presentations in children with current food allergy

Among children with current food allergy, the main ARFID‐FAA variant reported was sensory sensitivity (18/31), followed by FAA (16/31) (two participants had missing data and were therefore excluded). Even though sensory sensitivity was the most common variant, it tended to co‐occur with other variants. The most common ARFID presentation was the combined subtype *n* = 13/31, followed by fear (fear of choking, vomiting and/or food allergy anxiety) *n* = 12/31, sensory *n* = 5/31, and lack of interest *n* = 1/31.

## 4 DISCUSSION

The prevalence of possible ARFID was common in 10‐year‐old participants of the population‐based HealthNuts study (21%, 95% CI 19%–24%) and was similar between children with and without food allergy (23% and 21%). However, as the EDY‐Q does not capture fear of adverse reactions related to food allergy, inclusion of food allergy anxiety (FAA) as a fear‐based ARFID variant suggested that the prevalence of possible ARFID‐FAA could be as high as (one in three) 32% in children with food allergy.

It is important to note that the EDY‐Q is a screening tool, identifying children at risk of ARFID and is not intended to provide a definitive diagnosis as it has not been validated against the gold standard clinical diagnostic interview. Our primary possible ARFID criteria included participants across the weight spectrum aligning with the current DSM5 criteria. Because earlier criteria restricted classification to individuals who were underweight, prevalence estimates based on these outdated definitions likely represent substantial underestimates and exclude many individuals who may be at risk of ARFID. Consistent with this, we observed a higher prevalence of possible ARFID in our general population sample compared with previous child population studies using the EDY‐Q that applied an underweight restriction (2.4%–3.2%).^31^, ^33^ However, these results were similar to our alternative (subjective underweight) possible ARFID definition which showed a 5% prevalence of possible ARFID in 10 years old. Factorial validity found the EDY‐Q represents the distinct variants of ARFID which were derived from the GOS criteria (food avoidance emotional disorder, selective eating and functional dysphagia).^34^ As the items on fear of aversive consequences centre on functional dysphagia, the EDY‐Q may not capture those who display food avoidance based on other food phobias such as fear of contamination, gastrointestinal upset and fear of allergic reactions. It is important to consider FAA as a variant of ARFID because it can encompass eating‐related behavior changes synonymous with ARFID. When FAA was incorporated into our ARFID definition, an additional 10 children with current food allergy were identified, bringing the prevalence of possible ARFID‐FAA to 32%, thus the EDY‐Q may not be a suitable tool for ARFID‐screening in children with food allergy.

There have since been validated diagnostic interviews and questionnaires developed for ARFID.^35^, ^36^ The Pica, ARFID, and Rumination Disorder Interview ARFID Questionnaire (PARDI‐AR‐Q) is derived from the PARDI interview and was designed to assess ARFID diagnosis criteria rather than just the presence of ARFID variants.^37^ Importantly, the PARDI‐AR‐Q is not intended as a standalone measure as it doesn't assess ARFID exclusion criteria; therefore, (body image or weight/shape concerns which may be suggestive of other restrictive eating disorders), thus it should be used in conjunction with other tools such as the Eating Disorders Examination Questionnaire (EDE‐Q).^37^, ^38^ Although the PARDI‐AR‐Q includes items addressing broader aspects of fear of aversive consequences such as fear of allergic reactions, this subscale needs further validation as initial validation studies had small numbers of ARFID cases with this presentation.^38^, ^39^ While initial validation studies of the PARDI‐AR‐Q are promising, sensitivity within the food allergy population remains unclear.

Schoffel et al. 2021,^33^ used the EDY‐Q to assess ARFID symptoms across the weight spectrum in 7–18 year‐olds from a general population (recruited by the ‘Leipzig Research Centre for Civilization Diseases (LIFE)’ Child study) and a general inpatient pediatric clinical (common conditions included type I diabetes, gastrointestinal disorders, and neurological disorders). Like our findings, they found little evidence of a difference in possible ARFID between the general and clinical populations (14% (110 / 799) vs. 10% (11 / 111), *p* = .29). This could reflect that the EDY‐Q overlooks other fear‐ARFID subtypes, which may be important in clinical populations; however, more studies are needed to confirm this. A recent study of 1–18 yrs. referred to a multidisciplinary complex eosinophilic esophagitis (EOE) clinic found that a greater number of co‐occurring IgE‐mediated allergies was associated with higher ARFID prevalence.^40^ Compared with the only other study of ARFID in IgE‐mediated food allergy,^21^ we observed a lower prevalence of possible ARFID (32% vs. 63%). Patrawala et al.^21^ identified probable ARFID using psychologist‐conducted interviews based on DSM5 criteria among children with doctor‐diagnosed IgE‐mediated food allergy referred to a tertiary clinic. Importantly, the authors made a concerted effort to define and classify ARFID cases in the context of food allergy.^21^ Several factors may explain the discrepancy between findings including use of a convenience sample, recruitment from a specialist feeding clinic and a clinical food allergy population that may represent more severe or newly diagnosed allergy. There is a lack of accurate population prevalence data in Australia. Two Australian studies reported ARFID prevalence of ~2% in 1–19 year olds and 0.3% in those ≥15 years.^9^, ^41^ Both studies used the DSM5 definition of ARFID; however, excluded individuals who avoid food for medical reasons including food allergy, potentially underestimating prevalence.

Our findings show one in five 10‐year‐olds screen positive for being at risk of ARFID, suggesting it is a common problem. However, some studies suggest that clinicians may be unfamiliar with ARFID, highlighting a potential gap in appropriate recognition and referral.^42^, ^43^, ^44^, ^45^ A recent EACCI task force review^46^ identified a wide range of overlapping terms currently used to describe feeding difficulties, including ARFID, within food allergy populations. It is important to distinguish between adaptive vigilance required for allergy management from FAA or other defined anxiety or phobia disorders, as well as from situations in which anxiety and/or phobia symptoms lead to restrictive eating and subsequent ARFID manifestations. Others have reviewed the literature on anxiety in food allergy and characterized the features of FAA.^4^, ^47^ FAA can occur without disordered eating or excessive dietary restriction. However, when food restriction or avoidance is associated with growth, nutrition or psychosocial compromise beyond that attributed to routine food allergy management, an ARFID diagnosis could be considered.^48^ In the context of food allergy, differential diagnosis such as parental anxiety, low nutrition/food/health literacy, undiagnosed or active eosinophilic disorders and food allergy‐related social anxiety/phobias should also be considered.^49^ As our study used the EDY‐Q which only screens for ARFID variants, we cannot distinguish children who may have FAA without ARFID; therefore, we have not been able to quantify the overlap between FAA and an ARFID diagnosis.

Key strengths of this study include robust, gold standard measures of IgE‐food allergy, the use of an ARFID screening tool with psychometric validation and that the HealthNuts sample at recruitment has demonstrated good external validity to the wider population.^22^ Our study had several limitations. The HealthNuts cohort at age 1 year was representative of the target population of infants in Melbourne, Australia; however, attrition by the 10‐year follow‐up has led to over‐representation of participants with a family or personal history of allergic disease.^1^ Our analytic sample comprised a subset of the 10‐year participants because the EDY‐Q was added part way through the study. Although children eligible for the EDY‐Q were more likely to have a history of food allergy, participants were otherwise comparable across demographic characteristics. As eligibility for the EDY‐Q was determined solely by timing of follow‐up, with no additional selection criteria, this difference likely represents chance imbalance rather than systematic selection. Nonetheless, we cannot exclude the possibility that temporal trends in food allergy diagnosis or other unmeasured factors associated with follow‐up timing could have influenced these differences. The EDY‐Q was the only available ARFID screening tool for our target age at the time of data collection. Using the EDY‐Q as originally designed limits cases to individuals with low weight. Removing the underweight requirement increases the sensitivity of ARFID screening but may reduce specificity, potentially identifying children who display ARFID variants but who otherwise may not meet full ARFID diagnostic criteria. Additionally, the EDY‐Q captures ARFID symptoms without requiring demonstrated restriction. For instance, EDY‐Q item 10 (“*I am afraid of choking or vomiting while eating*”) does not specify associated food avoidance, likely contributing to overestimation. FAA questions from the FAQLQ‐PF were parent‐reported and not validated for use in isolation; this questionnaire was administered to those with reported food allergy therefore we could not estimate ARFID‐FAA in those without food allergy as a comparison. Additionally, as there are no validated clinical cut‐off scores for the FAQL questionnaires, our cut‐off for the FAA item should be interpreted as study‐defined cut‐off rather than a clinical standard. Consequently, ARFID‐FAA is an exploratory definition and future studies are needed to validate this construct.

## 5 CONCLUSION

A similar proportion of children with and without food allergy were identified by the EDY‐Q of being at risk of ARFID. Possible ARFID was high in children with and without food allergy. The EDY‐Q may lack sensitivity to detect ARFID in individuals with food allergy as it does not capture fear of allergic reactions. Newer ARFID diagnostic tools incorporating fear of allergic reactions have since become available. Future research should prioritize validation of these tools in food allergy cohorts using the gold‐standard clinical interview as the reference. This will guide the use of screening tools in allergy clinics and provide more precise prevalence estimates and, importantly, enable identification of children who may benefit from tailored ARFID management strategies.

## AUTHOR CONTRIBUTIONS

**Michele Yeo:** Supervision; writing – review and editing. **Rushani Wijesuriya:** Writing – review and editing; methodology. **Mimi L. K. Tang:** Writing – review and editing; funding acquisition. **Jennifer J. Koplin:** Supervision; methodology; data curation; writing – review and editing; funding acquisition. **Kirsten P. Perrett:** Writing – review and editing. **Daniela Ciciulla:** Conceptualization; writing – original draft; formal analysis; methodology; writing – review and editing. **Rachel L. Peters:** Supervision; writing – review and editing; methodology. **Vicki McWilliam:** Supervision; writing – review and editing.

## FUNDING INFORMATION

This work was supported by funding from the National Health and Medical Research Council (NHMRC) of Australia, Ilhan Food Allergy Foundation, AnaphylaxiStop, the Charles and Sylvia Viertel Medical Research Foundation, and the Victorian Government’s Operational Infrastructure Support Program. D.C. is supported by PhD funding from the NHMRC funded Centre for Food Allergy Research (CFAR) and by the Commonwealth through an Australian Government Research Training Program Scholarship [DOI: https://doi.org/10.82133/C42F‐K220]. DC received a Henry and Rachel Ackman Traveling Scholarship and a CFAR Travel grant to present this work. R.L.P., J.J.K. and K.P.P. receive research support from NHMRC. V.M. and K.P.P. are supported by a Melbourne Children’s Clinician–Scientist Fellowship. K.P.P. is also supported by a NHMRC fellowship, GNT2008911.

## CONFLICTS OF INTEREST STATEMENT

K.P.P. has received research grants from Aravax, DBV Technologies, Novartis and Siolta and consultant fees from Aravax, Novartis and RAPT Therapeutics, paid to their institution, outside the submitted work. Research at the Murdoch Children’s Research Institute is supported by the Victorian Government’s Operational Infrastructure Program. R.L.P. has received research funding from the NHMRC, National Peanut Board, Asthma Australia, Thoracic Society of Australia and New Zealand (TSANZ), and Allergy and Immunology Foundation of Australasia (AIFA); a prize from the Stallergenes Greer Foundation; and speaker fees from Thermo Fisher Scientific, all paid to their institution outside of the submitted work. J.J.K. has received a prize from the Stallergenes Greer Foundation paid to her institution outside of the submitted work. M.L.K.T. declares consultant fees from Pfizer and CSL Seqirus; holds shares/options in Prota Therapeutics; is a member of the Medical Advisory Board of Allergy & Anaphylaxis Australia; is a member of the Board of Directors of Asia Pacific Association of Allergy Asthma and Clinical Immunology, AllergyPal and Prota Therapeutics; is a member of expert committees of the American Academy of Allergy, Asthma & Immunology, Asia Pacific Association of Allergy Asthma and Clinical Immunology, Australasian Society of Clinical Immunology and Allergy, and World Allergy Organization. Other authors declare no conflicts of interest.

## Supporting information


**Table S1.** Baseline characteristics between participants and non‐participants of the child questionnaire.
**Table S2.** Sensitivity analysis with different ARFID definitions.
**Table S3.** Distribution of possible ARFID by demographic and clinical characteristics among children with history of food allergy ever.

## Data Availability

The data that support the findings of this study are available from the corresponding author upon reasonable request.
