# Supplementary material for: Prevalence of Avoidant/Restrictive Food Intake Disorder (ARFID) in children with and without food allergy
Source: Pediatr Allergy Immunol. 2026 Jul 7;37(7):e70393. doi: 10.1111/pai.70393 (PMC13342918; doi:10.1111/pai.70393)
Supplement: Supplementary file 1 — Table S1. Baseline characteristics between participants and non‐participants of the child questionnaire. Table S2. Sensitivity analysis with different ARFID definitions. Table S3. Distribution of possible ARFID by demographic and clinical characteristics among children with history of food allergy ever. [file PAI-37-e70393-s001.docx]

**Supplementary**

Supp Table 1.Baseline characteristics between participants and non-participants of the child questionnaire

| **Factors at age 1** | **Child Questionnaire non-participants**    **N=2992** | **Child Questionnaire but no EDY-Q**  **N=1322** | **EDY-Q**    **N=952** | **Whole cohort**        **N=5276** |
| --- | --- | --- | --- | --- |
| Sex - male, n (%)  Missing | 1495 (50%)  20 | 680 (52%)  9 | 487 (51%)  2 | 2665 (51%)  32 |
| SES at age 1, n (%)  1 (most disadvantaged)  2  3  4  5 (least disadvantaged)  Missing | 657 (22%)  575 (19%)  607 (20%)  577 (19%)  565 (19%)  11 | 210 (16%)  287 (22%)  286 (22%)  253 (19%)  285 (22%)  1 | 193 (20%)  187 (20%)  207 (22%)  189 (20%)  175 (18%)  1 | 1062 (20%)  1050 (20%)  1102 (21%)  1019 (19%)  1028 (20%)  15 |
| Parental country of birth, n (%)  Both born Australia  One / both born south / east Asia  One / both born United Kingdom Europe  Other  Missing | 1670 (57%)  478 (16%)  393 (13%)  387 (13%)  64 | 788 (63%)  154 (12%)  167 (13%)  150 (12%)  63 | 598 (63%)  150 (16%)  109 (11%)  94 (10%)  1 | 3060 (59%)  785 (15%)  670 (13%)  631 (12%)  130 |
| Family history of allergy, n (%)  Missing | 2008 (67%)  - | 959 (73%)  - | 689 (72%)  - | 3661 (70%)  - |
| Family history of food allergy, n (%)  Missing | 292 (10%)  59 | 155 (12%)  24 | 130 (14%)  25 | 577 (11%)  108 |
| Any food allergy at age 1, n (%)  Missing | 217 (8%)  262 | 165 (13%)  62 | 151 (17%)  43 | 534 (11%)  369 |

**Supp Table 2** – **Sensitivity analysis with different ARFID definitions.**

|  | *ARFID primary definition  % (CI)* | *ARFID subjective underweight  % (CI)* |
| --- | --- | --- |
| *Total N* | 204 / 951 | 43 / 951 |
| *Food allergy ever*  *No (n=767)*  *Yes (n=184)* | 21% (18 – 24)  23% (17 – 29) | 4% (3 – 6)  6% (3 – 10) |
| *Current food allergy*  No (n = 771)  Yes (n = 102) | 21% (18 – 24)  23% (15 – 32) | 4% (3 – 6)  7% (3 – 14) |
| *Change in egg and peanut allergy over time*  Never egg allergic (n=632)  Resolved egg allergy (n=109)  Persistent egg allergy (n=12)  Never peanut allergic (n=693)  Resolved peanut allergy (n=12)  Persistent peanut allergy (n=55) | 22% (19 – 25)  23% (16 – 32)  25% (7 – 60)  22% (19 – 25)  17% (4 – 52)  20% (11 – 33) | 4% (3 – 5)  8% (4 – 15)  8% (1 – 5)  4% (3 – 6)  0  4% (1 – 14) |
| Anaphylaxis ever  Never food allergy (n= 767)  Food allergy ever without anaphylaxis (n=83)  Food allergy ever with anaphylaxis (n=67) | 21% (18 – 24)  21% (14 – 32)  23% (15 – 35) | 4% (3 – 6)  4% (1 – 11)  7% (3 – 16) |
| Adrenaline autoinjector prescription ever  Never food allergy (n= 767)  Food allergy ever without prescription (n=94)  Food allergy ever with prescription (n=90) | 21% (18 – 24)  24% (17 – 34)  21% (14 – 31) | 4% (3 – 6)  5% (2 – 12)  7% (3 – 14) |

**Supp Table 3.** Distribution of possible ARFID by demographic and clinical characteristics among children with history of food allergy ever

|  | **Food Allergy EVER** | |  |
| --- | --- | --- | --- |
|  | **No ARFID N=142** | **Possible ARFID N=42** | **P value** |
| Sex  Male  Female | 93 (78%)  49 (77%) | 27 (23%)  15 (23%) | 0.79 |
| SES score^+^  1 (most disadvantaged)  2  3  4  5 (least disadvantaged) | 18 (69%)  31 (89%)  34 (79%)  25 (69%)  34 (77%) | 8 (31%)  4 (11%)  9 (21%)  11 (31%)  10 (23%) | 0.31 |
| Ethnicity  Both parents Caucasian  One or both parents Aboriginal or Torres strait Islander  One or both parents Asian  Other | 84 (80%)  1 (50%)  49 (75%)  8 (67%) | 21 (21%)  1 (50%)  16 (25%)  4 (33%) | 0.55 |
| Parental country of birth Both born Australia  One or both born south / east Asia  One or both born United Kingdom Europe  Other | 72 (77%)  39 (80%)  12 (80%)  18 (69%) | 21 (23%)  10 (20%)  3 (20%)  8 (31%) | 0.76 |
| Family history of allergy  No  Yes | 31 (82%)  111 (76%) | 7 (18%)  35 (24%) | 0.47 |
| Height z score, (mean SD)  Missing | 0.3 (1.0)  5 | 0.4 (1.0)  3 | 0.75 |
| BMI z scores (mean (SD))  *Missing* | 0.1 (1.0)  *7* | -0.2 (1.1)  *3* | 0.24 |
| BMI percentile  < 5^th^ Percentile  5-85^th^ Percentile  85-95^th^ Percentile  >95^th^ Percentile | 3 (60%)  103 (76%)  17 (100%)  12 (75%) | 2 (40%)  33 (24%)  0  4 (25%) | 0.11 |
| *Deceleration in BMI z score from age 6yrs to 10yrs*  *No deceleration*  *-1 z score*  *-2 z scores*  *-3 scores* | 119 (78%)  7 (70%)  *0*  *1 (100%)* | 32 (21%)  3 (30%)  *0*  *0* | 0.70 |
| Subjective underweight  No  Yes | 121 (80%)  20 (65%) | 31 (20%)  11 (35%) | 0.069 |
| Current allergic comorbidities  No  Yes | 31 (84%)  101 (76%) | 6 (16%)  32 (24%) | 0.31 |
| Current Eczema  No  Yes | 86 (77%)  37 (74%) | 26 (23%)  13 (26%) | 0.70 |
| Current Asthma No  Yes | 109 (79%)  33 (72%) | 29 (21%)  13 (28%) | 0.31 |
| Current Hay fever  No  Yes | 63 (85%)  76 (74%) | 11 (15%)  27 (26%) | 0.07 |
| Anaphylaxis ever  No  Yes | 65 (78%)  53 (77%) | 18 (22%)  16 (23%) | 0.83 |
| Adrenaline Autoinjector prescription ever  No  Yes | 71 (76%)  71 (79%) | 23 (25%)  19 (21%) | 0.59 |

^+^ SES score collected at wave 1 (12months old). ^^^Includes hx of Food allergy, asthma, eczema and hay fever in parents and siblings collected at wave 1 (12months old). ^^^^Any current Eczema, Asthma or hay fever; The definitions of current eczema, asthma and hay fever have been previously described by Peters et al^1^

References.

1. Peters RL, Soriano VX, Allen KJ, Tang ML, Perrett KP, Lowe AJ, et al. The prevalence of IgE-mediated food allergy and other allergic diseases in the first 10 years: the population-based, longitudinal HealthNuts study. The Journal of Allergy and Clinical Immunology: In Practice. 2024;12(7):1819–30. e3.
